# Oral and Fecal Microbiota in Lynch Syndrome

**DOI:** 10.3390/jcm9092735

**Published:** 2020-08-24

**Authors:** Roberto Ferrarese, Raffaella Alessia Zuppardo, Marta Puzzono, Alessandro Mannucci, Virginia Amato, Ilaria Ditonno, Maria Grazia Patricelli, Annalisa Russo Raucci, Massimo Clementi, Ugo Elmore, Riccardo Rosati, Pier Alberto Testoni, Nicasio Mancini, Giulia Martina Cavestro

**Affiliations:** 1 Microbiology and Virology Unit, IRCCS Ospedale San Raffaele Scientific Institute, 20132 Milan, Italy; ferrarese.roberto@hsr.it (R.F.); amato.virginia@hsr.it (V.A.); clementi.massimo@hsr.it (M.C.); mancini.nicasio@hsr.it (N.M.); 2 Division of Experimental Oncology, Gastroenterology and Gastrointestinal Endoscopy Unit, IRCCS Ospedale San Raffaele Scientific Institute, 20132 Milan, Italy; zuppardo.raffaellaalessia@hsr.it (R.A.Z.); puzzono.marta@hsr.it (M.P.); testoni.pieralberto@hsr.it (P.A.T.); 3 Vita-Salute San Raffaele University, 20132 Milan, Italy; alessandromannucci11@gmail.com (A.M.); ilariaditonno@gmail.com (I.D.); rosati.riccardo@hsr.it (R.R.); 4 Division of Genetics and Cell Biology and Laboratory of Clinical Molecular Biology and Cytogenetics, Unit of Genomics for Human Disease Diagnosis, IRCCS Ospedale San Raffaele Scientific Institute, 20132 Milan, Italy; patricelli.mariagrazia@hsr.it (M.G.P.); russoraucci.annalisa@hsr.it (A.R.R.); 5 Department of Gastrointestinal Surgery, IRCCS Ospedale San Raffaele Scientific Institute, 20132 Milan, Italy; elmore.ugo@hsr.it

**Keywords:** lynch syndrome, gut microbiota, oral microbiota, colorectal neoplasms

## Abstract

Background: The role of microbiota in Lynch syndrome (LS) is still under debate. We compared oral and fecal microbiota of LS saliva and stool samples with normal healthy controls (NHC). Methods: Total DNA was purified from feces and saliva to amplify the V3–V4 region of the 16s rRNA gene. Sequences with a high-quality score and length >250 bp were used for taxonomic analysis with QIIME software. Results: Compared to NHC, LS fecal samples demonstrated a statistically significant increase of Bacteroidetes and Proteobacteria and a significant decrease of Firmicutes at the phylum level and of Ruminococcaceae at the family level. Moreover, LS oral samples exhibited a statistically significant increase of Veillonellaceae and Leptotrichiaceae and a statistically significant decrease of Pasteurellaceae. A beta-diversity index allowed differentiation of the two groups. Conclusions: A peculiar microbial signature is associated with LS, similar to that of sporadic colorectal cancer and Crohn’s disease. These data suggest a possible role of proinflammatory bacteria in tumor development in a condition of genetic predisposition, such as LS.

## 1. Introduction

Lynch syndrome (LS) is an autosomal dominant hereditary cancer syndrome with variable penetrance and expressivity [1]. LS accounts for 3% of unselected colorectal cancers (CRCs) and 2% of endometrial cancers, and estimates suggest that as many as one in every 270 people may be carriers of LS [2,3]. LS is caused by pathogenetic variants in *MLH1*, *MSH2*, *MSH6*, and *PMS2* genes, which guarantee DNA mismatch repair (MMR). Failure of the MMR system provokes generalized genome instability, especially in the short repetitive coding and noncoding sequences [1]. Patients with LS develop less aggressive forms of CRC, and they overall have a better prognosis than patients with sporadic CRCs. Neoplasms from patients with LS have distinctive histological characteristics, such as a local inflammatory response [4] with abundant tumor-infiltrating T-lymphocytes and a Crohn’s-like reaction. The higher density of tumor-infiltrating leukocytes may account for the better prognosis, compared to sporadic CRCs, which generally lack cytotoxic T-lymphocytes. Indeed, the evasion of immune destruction is considered one of the hallmarks of cancer, and the tumor-infiltrating leukocytes may represent an attempt to mount an antitumor immune response [5,6].

Tumors from patients with LS express numerous neoepitopes, which could elicit an antigen-specific cytotoxic T-cell response [7,8,9]. Tumor cells, incapable of DNA repair, accumulate somatic mutations in protein-coding genes. Intracellular processing converts aberrant proteins into immunogenic peptides—neoantigens—that bind HLA Class I, activate naïve CD8+ T-cells, and transform them into cytotoxic T-cells, capable of mediating tumor lysis [10,11,12]. One hypothesis is that this proinflammatory milieu could change the microbiota in terms of the overall composition, diversity, and taxonomic pattern abundance, thus increasing the risk of developing CRC [13]. As a result, in LS, the consequences of a genetically determined, site-specific, altered MMR system might be worsened by nongenetic factors, including microbiota.

Changes to the microbiota contribute to sporadic colorectal carcinogenesis [14]. An increase in Fusobacterium nucleatum often precedes intestinal dysbiosis [15]. This promotes inflammatory changes [16], alterations in DNA stability [17], modulation of E-cadherin/β-catenin signaling [18], and overexpression of FadA and fap2, which directly target carcinogenic pathways [16,18,19]. Experimental evidence from mouse models suggests that different community compositions can result in a diverse tumor burden [20,21]. Moreover, the dysplastic transformation [22,23] and the adenoma–carcinoma process [24] alter the gut microbiome, but after treatment, the intestinal flora reverts to a healthy one [25]. A recent study indicated a connection between alterations in DNA methylation, microbiota composition, and CRC. Germ-free mice receiving fresh feces from CRC patients developed colon epithelial proliferation, precancerous lesions, and increased DNA methylation in intestinal tissue and blood [26].

The contribution of the microbiota to colorectal carcinogenesis in patients with LS is relatively unknown. Yan et al. recently profiled the microbiota from fecal and histological specimens of 100 LS patients at baseline and a one-year follow-up, and they observed that dysbiosis developed with colonic preneoplastic lesions (e.g., adenomas). A colectomy can change the overall microbiome structure. Nonetheless, baseline differences in mucosal and fecal community function were concordant with previously observed changes in later-stage CRC and weakly predictive of interval adenoma development [27]. Moreover, LS patients with CRC had fecal microbial communities similar to those from LS patients with gynecological tumors [28].

We aim to characterize the salivary and fecal microbial population of LS patients compared to healthy controls. We are specifically interested in investigating the overall microbiota composition, diversity, and taxonomic pattern abundance in LS patients.

## 2. Experimental Section

### 2.1. Patients and Sample Collection

Patients were consecutively enrolled from 2017 to 2019 at IRCCS San Raffaele Scientific Institute, a tertiary referral hospital in Milan, Italy. Patients with LS had their DNA sequenced with a Next Generation Sequencing (NGS) panel that included the 4 MMR genes and the *EPCAM* gene: class IV and class V variants were collectively considered to be pathogenic, according to the InSiGHT criteria by Thomson et al. [29].

Seventeen patients with LS provided fecal samples (13 females and four males, mean age 48 ± 15,8): two female subjects with an *MLH1* pathogenetic variant, 14 subjects with an *MSH2* pathogenetic variant (10 females and four males), and one female subject with an *MSH6* pathogenetic variant. Thirty-seven patients provided salivary samples (28 females and nine males, mean age 56 ± 34,28): nine subjects with an *MLH1* pathogenetic variant (eight female and one male), 23 subjects with an *MSH2* pathogenetic variant (15 female and eight male), two female subjects with an *MSH6* pathogenetic variant, and three female subjects with a *PMS2* pathogenetic variant. Four *MLH1* patients, 10 *MSH2* patients, one *MSH6* patient, and one *PMS2* patient had undergone hemicolectomy for CRC: nine had received (two *MLH1*, seven *MSH2*) a right hemicolectomy and seven (two *MLH1*, three *MSH2*, one *MSH6*, one *PMS2*) a left hemicolectomy. Two patients (one *MLH1*, one *MSH2*) received a gastrectomy for gastric cancer. Two patients had a pancreatic resection: one *MSH6* patient for pancreatic cancer, and one *PMS2* patient for a neuroendocrine tumor.

Healthy normal control subjects were tested for their likelihood of harboring a mutation in the MMR genes, employing PREMM5 predictive Model (Dana-Farber Cancer Institute, Inc., Boston, MA 02215, USA) [30]. The study excluded individuals with a PREMM5 of 2.5% or higher from the control cohort, who instead received genetic counseling. The study did not consider eligible to the control cohort individuals who had any cancer, inflammatory bowel diseases, metabolic syndrome, who had used antibiotics in the 15 days before, or who used chronic medication. The study included individuals who had no family history for cancers, with PREMM5 < 2.5%, and negative past and recent clinical history.

Healthy, age-matched controls provided 11 salivary samples (mean age 54 ± 3.77, all females) and 21 fecal samples (17 female and four males, mean age 52.5 ± 6.9).

Salivary samples were retrieved with an oral wash with sterile physiological solution and immediately frozen at −80.0 °C. Patients received instructions on how to collect their fecal samples: within seven days of their upcoming visit, they sampled a small volume of feces in a 0.5 mL Eppendorf tube that contained RNAprotect Tissue Reagent (QIAGEN, Venlo, The Netherlands). They kept fecal samples in their freezers at home and then brought them to the clinic using iceboxes. LS patients and normal controls had to be completely healthy in the three months before sampling, without using antibiotics or anti-inflammatory drugs. All LS patients were cancer-free at the time of sample collection and analysis. Patients on chemotherapy could not participate in the study. On average, 9.2 years had elapsed from previous surgery and chemotherapy to the day of sample collection. Based on findings from Feng et al. [24], this guarantees that the oncological treatment did not alter the microbiota analysis.

This study conformed to the ethical guidelines of the 1975 Declaration of Helsinki (6th revision, 2008), and it was reviewed and approved by the Institutional Review Board of IRCCS Ospedale San Raffaele on 12 July 2010 (protocol: BIOGASTRO/2011, ver.2 of 17 October 2013, Milan, Italy). Patients and controls both provided written informed consent for study participation.

### 2.2. Microbiota Analysis

The microbiota analysis was performed by 16S amplicon sequencing. Total DNA was purified from fecal and oral samples using the QIAamp PowerFecal DNA Kit and the Dneasy Blood & Tissue Kits (QIAGEN, Venlo, The Netherlands), following the manufacturer’s instructions. The V3–V4 region of the 16S rRNA gene was amplified starting from 200 ng of extracted DNA using the AccuPrime Taq DNA Polymerase (Invitrogen, Waltham, MA, USA), the following primers: V3-16S-Fw: CCT ACG GGN GGC WGC AG and V4-16S-Rev: GAC TAC HVG GGT ATC TAA TCC, and the following amplification protocol: 94 °C for 2 min, 35 cycles of 94 °C for 30 s, 56 °C for 30 s, 68 °C for 1 min, and finally stored at 4 °C. Amplicons were purified using AMPure XP beads (Beckman Coulter, Brea, CA, USA). A second PCR step was performed for indexing and adding Illumina sequencing adapters to each sample. The Nextera XT Index Kit (Illumina, San Diego, CA, USA) and the KAPA HiFi HotStart PCR Kit (KAPA Biosystem, Basel, Switzerland) were used with the following amplification protocol: 95 °C for 3 min, 8 cycles of 95 °C for 30 s, 55 °C for 30 s, 72 °C for 30 s, 72 °C for 4 min, and then stored at 4 °C. A second purification step with AMPure XP beads was performed to clean up the final library. The purified DNA was quantified using a Qubit Fluorometer (Thermo Fisher, Waltham, MA, USA) and a 2100 Bioanalyzer System (Agilent, Santa Clara, CA, USA). Libraries were diluted and mixed for pooling with unique molecular tags. The pool was loaded on a MiSeq reagent cartridge. Sequences with a high-quality score and length >250 bp were used for the taxonomic analysis with Quantitative Insights Into Microbial Ecology v1.9.1 software (QIIME [31]).

### 2.3. Statistical Analysis

The analysis excluded less abundant bacterial taxa, <1% in all samples, at any taxonomic level. Bacterial relative abundance was reported as an average ± standard error percentage. The statistical significance of differences in means and proportions among LS patients and controls was tested with Welch’s *t*-test with Benjamini–Hochberg FDR multiple test correction. To determine if factors like sex and age had an impact on the statistical outcome, we performed a binomial logistic regression using the healthy/pathological status as the dependent variable and the relative abundance of bacteria, age, and sex as the independent variables. Weighted UniFrac distance metric and principal component analysis were used to perform the beta-diversity analysis: the Adonis statistical method was used to calculate the differences in beta diversity between the groups, describing the strength and significance that a variable has in determining the variation of distances in a beta-diversity graph. Microsoft Office Excel 2010™ (Microsoft, Redmond, WA, USA) and Graphpad Prism 5™ (GraphPad Software, SD, USA) were used to perform statistical tests. All tests were two-sided, with a significance level set at 0.05.

## 3. Results

We analyzed fecal samples from 17 LS patients and 21 normal controls and oral samples from 37 LS patients and 11 normal controls. The analysis excluded groups at any taxonomic level representing <1%.

**Figure 1:**
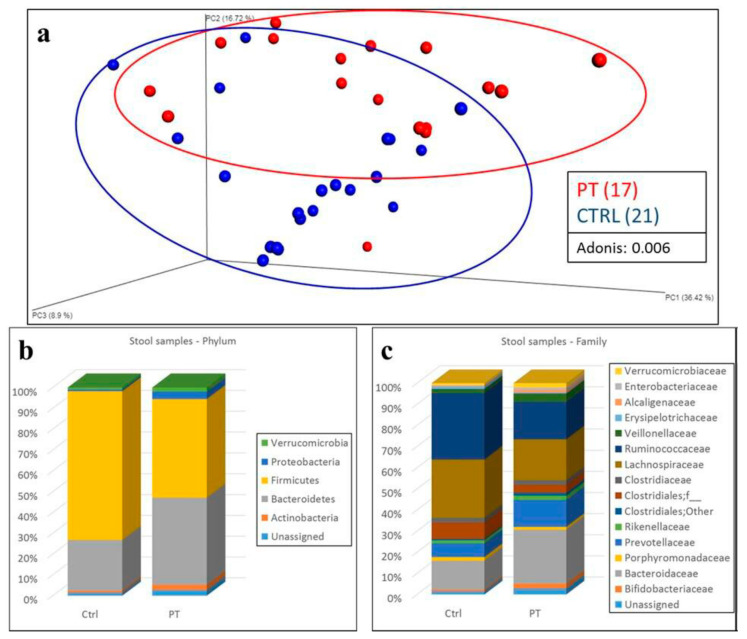
Analysis of fecal samples. (**a**) Beta diversity analysis of fecal samples with respect to subject status: Lynch syndrome patients = PT (red); control subjects = CTRL (blue). Weighted UniFrac distance metric and principal component analysis were used to perform beta-diversity analysis. A cluster can be observed between the two groups, confirmed by Adonis analysis (*p*: 0.006). Statistical analysis: Adonis. Statistical significance: *p* < 0.05. (**b**,**c**) Taxonomic composition of stool samples from Lynch syndrome patients (PT) and control subjects (CTRL) at phylum (**b**) and family (**c**) levels. Relative bacterial abundances are expressed as average percentage. Statistical significance: *p* < 0.05.

In fecal samples, alpha diversity—the variation of microbes in a single sample—had no statistically significant difference between the two cohorts. Operational taxonomic units (OTUs) and the Shannon index demonstrated lower values among LS patients, but without reaching statistical significance (Figure S1). Beta diversity analysis, the variation of microbial communities between samples, performed using the weighted UniFrac distance metric and principal component analysis, allowed us to distinguish the two populations of LS patients and control subjects (Figure 1a).

Analysis of the microbiota composition revealed significant differences between patients and controls according to the relative abundance of bacteria. We identified 21 bacterial phyla, and five had an average relative abundance >1%: Bacteroidetes, Firmicutes, Proteobacteria, Actinobacteria, and Verrucomicrobia. Patients with LS had a statistically significant increase of Bacteroidetes (41.7% ± 2.6% vs. 24.5% ± 3.6%; q < 0.001) and Proteobacteria (3.5% ± 0.8% vs. 0.8% ± 0.2%; q = 0.029) and a decrease of Firmicutes (47.1% ± 2.8% vs. 71.2% ± 3.6%; q < 0.001) compared to control subjects (Figure 1b, Table S1). At the family level, we identified 128 bacterial families, 15 of which had an average bacterial relative abundance >1%: Bifidobacteriaceae, Bacteroidaceae, Porphyromonadaceae, Prevotellaceae, Rikenellaceae, unclassified Clostridiales, Clostridiaceae, Lachnospiraceae, Ruminococcaceae, Veillonellaceae, Erysipelotrichaceae, Alcaligenaceae, Enterobacteriaceae, and Verrucomicrobiaceae. At this phylogenetic level, patients with LS demonstrated a statistically significant increase of Alcaligenaceae (1.1% ± 0.2% vs. 0.3% ± 0.1%; q = 0.023) and a decrease of Ruminococcaceae (16.9% ± 1.9% vs. 30.2% ± 1.8%; q = 0.024) compared to control subjects (Figure 1c, Table S2).

**Figure 2:**
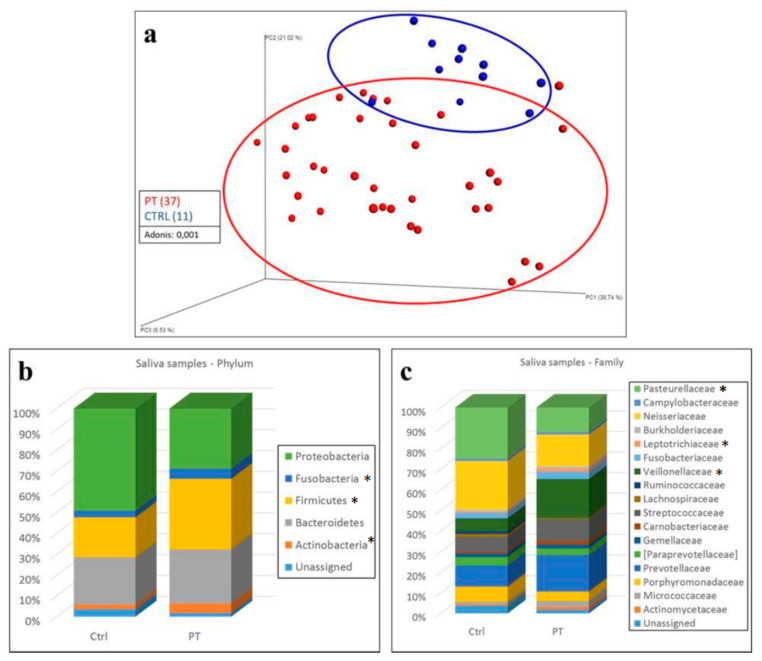
Analysis of salivary samples. (**a**) Beta diversity analysis of salivary samples with respect to subject status: Lynch syndrome patients = PT (red); control subjects = CTRL (blue). Weighted UniFrac distance metric and principal component analysis were used to perform beta-diversity analysis. A cluster can be observed between the two groups, confirmed by Adonis analysis (*p*: 0.001). Statistical analysis: Adonis. Statistical significance: *p* < 0.05. (**b**,**c**) Taxonomic composition of oral samples from Lynch syndrome patients (PT) and control subjects (CTRL) at phylum (**b**) and family (**c**) levels. Relative bacterial abundances are expressed as average percentage. * Statistical significance: *p* < 0.05.

In salivary samples, alpha diversity did not detect statistically significant differences between LS patients and control subjects in either observed OTUs or Shannon index analyses. However, patients with LS had lower values of alpha diversity (Figure S2). Beta diversity allowed us to distinguish LS patients from control subjects in this circumstance as well (Figure 2a).

As for fecal samples, analysis of the relative abundance of bacteria revealed statistically significant differences. There were 23 different phyla, and five had an average relative abundance >1%: Actinobacteria, Bacteroidetes, Firmicutes, Fusobacteria, and Proteobacteria. In LS patients, compared to control subjects, we observed a statistically significant increase of Actinobacteria (4.4% ± 1.2% vs. 2.1% ± 0.4%; q < 0.001) and Firmicutes (33.2% ± 2.1% vs. 18.7% ± 2.5%; q < 0.001) and a decrease of Proteobacteria (28.4 ± 2.7% vs. 48.2% ± 3.7%; q < 0.001) (Figure 2b, Table S3).

At the family level, we identified 156 families, 17 of which showed an average bacterial relative abundance >1%: Actinomycetaceae, Micrococcaceae, Porphyromonadaceae, Prevotellaceae, Paraprevotellaceae, Gemellaceae, Carnobacteriaceae, Streptococcaceae, Lachnospiraceae, Ruminococcaceae, Veillonellaceae, Fusobacteriaceae, Leptotrichiaceae, Burkholderiaceae, Neisseriaceae, Campylobacteraceae, and Pasteurellaceae. In particular, we observed in LS patient samples an increase of Veillonellaceae (18% ± 1.8% vs. 5.9% ± 2%; q < 0.001) and Leptotrichiaceae (1.3% ± 0.2% vs. 0.5% ± 0.2%; q = 0.026) and a decrease of Pasteurellaceae (11.4% ± 1.7% vs. 23.6% ± 2.3%; q < 0.001) compared to control samples (Figure 2c, Table S4).

We analyzed microbial abundance at the genus and species level in both fecal and saliva samples. Fecal samples from patients with LS were not significantly different from samples from controls (Tables S5 and S6) when tested with Welch’s *t*-test with Benjamini–Hochberg FDR multiple test correction. Conversely, salivary samples demonstrated statistically significant differences between groups at the genus level for the Veillonella (*p* < 0.01) and Haemophilus (*p* = 0.026) genera (Table S7), and at the species level for Veillonella dispar (*p* < 0.01) (Table S8; these were tested with Welch’s *t*-test with Benjamini–Hochberg FDR multiple test correction.

Binomial logistic regression confirmed the statistical significance of these findings, and it showed that age and sex did not influence the statistical outcome. Indeed, in fecal samples, there were statistically significant differences in the same bacterial taxa that were statistically different with Welch’s *t*-test with Benjamini–Hochberg FDR multiple test correction analysis (Phylum: Bacteroidetes, *p* = 0.004; Firmicutes, *p* = 0.004; Proteobacteria, *p* = 0.005. Family: Ruminococcaceae, *p* = 0.005; Alcaligenaceae, *p* = 0.001). No statistically significant differences were observed for age and sex variables. In saliva samples, there were statistically significant differences in the same bacterial taxa that were statistically different with Welch’s *t*-test with Benjamini–Hochberg FDR multiple test correction analysis (Phylum: Actinobacteria, *p* = 0.021; Firmicutes, *p* = 0.003; Proteobacteria, *p* = 0.004. Family: Veillonellaceae, *p* = 0.004; Leptotrichiaceae, *p* = 0.012; Pasteurellaceae, *p* = 0.004). No statistically significant differences were observed for age and sex variables.

## 4. Discussion

Lynch syndrome is an autosomal dominant cancer syndrome with variable penetrance and expressivity. LS results from pathogenetic variants in genes that guarantee DNA mismatch repair. This cross-sectional study details the microbiome composition of fecal and oral specimens from a cohort of patients with LS and compares it to that of age-matched healthy controls. Fecal samples from subjects with LS had a statistically significant increase of Bacteroidetes and Proteobacteria, a significant decrease of Firmicutes at the phylum level and of Ruminococcaceae at the family level, and a notable reduction of Lachnospiraceae (although not statistically significant). A significant decrease of Firmicutes was recently demonstrated in both stool samples and intestinal lavage fluid of CRC patients compared to controls [32]. Moreover, analogous variations occur in patients with Crohn’s disease, multiple sclerosis, and systemic lupus erythematosus [33]. While the increase of fecal Proteobacteria is able to enhance the permeability of the sterile inner layer of the intestinal mucus, resulting in bacterial inflammation close to the epithelium [34], the reduction of Lachnospiraceae and Ruminococcaceae is relevant because of the consequent decrease in butyrate production and its beneficial effects. Indeed, butyrate is an important energy source for intestinal epithelial cells as it alleviates mucosal inflammation, modulates visceral sensitivity and intestinal motility, and controls carcinogenesis [35]. Moreover, the evaluation of the oral microbiome has gained importance for its role in gastrointestinal health as a possible predictor of pathological conditions, including CRC [36,37,38,39].

The statistically significant differences in the oral and fecal microbiota of LS individuals, compared to normal healthy controls, would add to our understanding of the disease. Currently, three alternative models describe the development of MMR deficiency. One model hypothesizes that, initially, an adenoma forms through the usual mechanisms (*WNT* inactivation and biallelic *APC* loss), and then MMR deficiency occurs, prompting the transition to CRC. As evidence suggests, MMR deficiency in LS patients develops in adenomas that are larger than 8 mm [40]. However, other findings challenge this model and suggest that MMR deficiency may occur earlier. Sekine and colleagues [41] found MMR deficient adenomas without *APC* mutations, which implies that MMR deficiency can occur during adenoma formation. Kloor and colleagues [42] detected MMR-deficient crypts in the intestinal epithelium. These findings result in two alternative hypotheses, according to which MMR deficiency can either prompt the development of an adenoma [41] or occur in the absence of an oncogenic process [42]. The current understanding of LS-associated oncogenesis is incomplete, and it follows the observation that some individuals with LS might develop antibodies against frameshift neopeptides before any cancer occurs.

Such conflicting evidence could blend in a unitary theory. LS-associated neoplasms have a peculiar local inflammatory response [4] with abundant tumor-infiltrating T-lymphocytes [43]. Early MMR deficiency would produce frameshift neopeptides that are presented on the cell surface by HLA. These would trigger the adaptive immune system and mount an immune response with T- and B-lymphocytes, thus explaining the presence of antibodies in individuals who are free from cancer. Therefore, a local inflammatory reaction would be too modest to alter the mucosa itself, but sufficient to remodel the oral and intestinal microbiota, resulting in the changes observed in this study.

This study is the first on oral microbiota in patients with Lynch syndrome, and this is one major strength. On the other hand, the small sample size of LS patients and the female prevalence in both patient and control groups are limitations of the study.

## 5. Conclusions

This study describes a microbial signature associated with LS, characterized by a statistically significant increase of Bacteroidetes and Proteobacteria and a significant decrease of Firmicutes at the phylum level and of Ruminococcaceae at the family level in LS fecal samples, similar to that observed in sporadic CRC. These results suggest a possible role of proinflammatory bacteria in tumor development in patients with a genetic predisposition. Indeed, an increase of fecal Proteobacteria is able to enhance the permeability of the sterile inner layer of the intestinal mucus, resulting in bacterial inflammation close to the epithelium [34]. Moreover, the reduction of Lachnospiraceae and Ruminococcaceae is associated with a decrease in butyrate production and its beneficial effects. These data need confirmation from larger cohorts of patients.

## Supplementary Materials

The following are available online at https://www.mdpi.com/2077-0383/9/9/2735/s1. Figure S1: Alpha diversity analysis in fecal samples. Figure S2: Alpha diversity analysis in salivary samples. Table S1: Differences in microbial abundance at the phylum level in fecal samples. Table S2: Differences in microbial abundance at the family level in fecal samples. Table S3: Differences in microbial abundance at the phylum level in saliva samples. Table S4: Differences in microbial abundance at the family level in saliva samples. Table S5: Differences in microbial abundance at the genus level in fecal samples. Table S6: Differences in microbial abundance at the species level in fecal samples. Table S7: Differences in microbial abundance at the genus level in saliva samples. Table S8: Differences in microbial abundance at the species level in saliva samples.
