# Supplementary material for: Oral and Fecal Microbiota in Lynch Syndrome
Source: J Clin Med. 2020 Aug 24;9(9):2735. doi: 10.3390/jcm9092735 (PMC7563889; doi:10.3390/jcm9092735)
Supplement: Supplementary file 1 [file jcm-09-02735-s001.pdf]

SUPPLEMENTARY MATERIALS

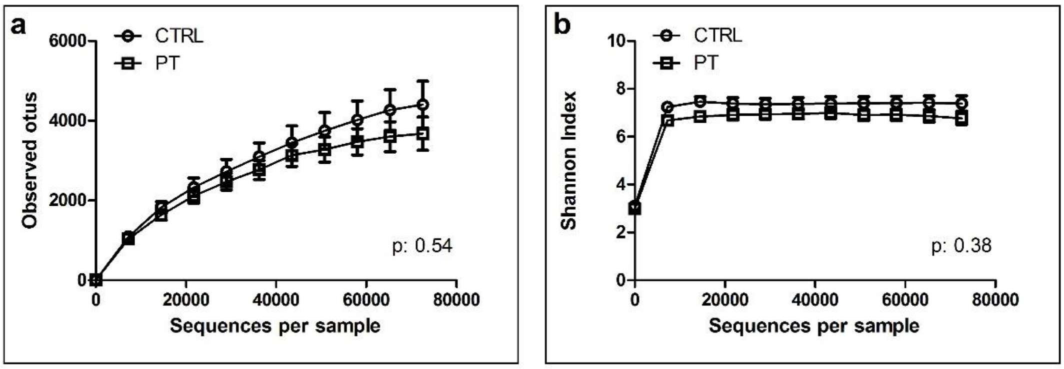

Supplementary Figure 1. Alpha diversity analysis in fecal samples. Diversity within groups (α-diversity), estimated as observed OTUs (a) and Shannon index (b) was assessed using >60.000 sequences per sample in Lynch syndrome patients (PT) and control subjects (CTRL). No statistically significant differences were observed. Statistical analysis: unpaired t-test.

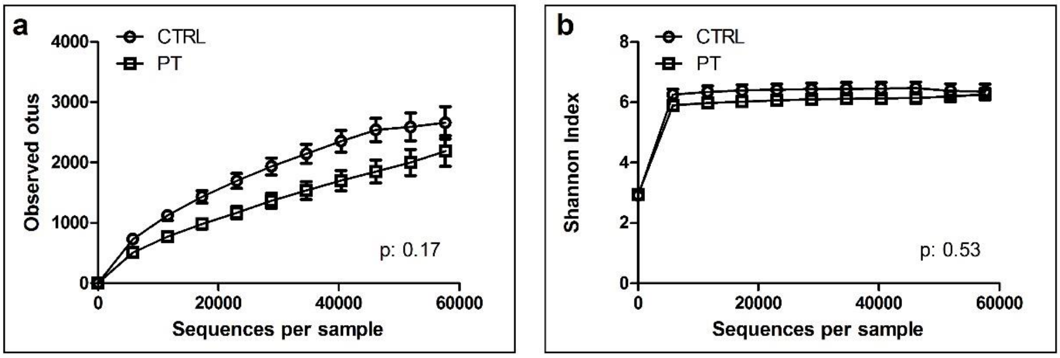

Supplementary Figure 2. Alpha diversity analysis in saliva samples. Diversity within groups (α-diversity), estimated as observed OTUs (a) and Shannon index (b) was assessed using >50.000 sequences per sample in Lynch syndrome patients (PT) and control subjects (CTRL). No statistically significant differences were observed. Statistical analysis: unpaired t-test.

Supplementary Table 1. Differences in microbial abundance at the phylum level in fecal samples. Statistical analysis: Welch's t-test with Benjamini-Hochberg FDR multiple test correction.

	Control Subjects	Lynch Patients	p value	q value
Unassigned bacteria	1,39%	2,71%	0,003	0,027
Actinobacteria	0,93%	2,59%	0,034	0,162
Bacteroidetes	24,48%	41,69%	0,001	<0,001
Firmicutes	71,22%	47,14%	<0,001	<0,001
Proteobacteria	0,81%	3,52%	0,005	0,029
Verrucomicrobia	1,00%	2,02%	0,604	0,659

Supplementary Table 2. Differences in microbial abundance at the family level in fecal samples. Statistical analysis: Welch's t-test with Benjamini-Hochberg FDR multiple test correction.

	Control Subjects	Lynch Patients	p value	q value
Unassigned bacteria	1,39%	2,71%	0,003	0,149
Bifidobacteriaceae	0,76%	2,16%	0,064	0,363
Bacteroidaceae	13,27%	24,34%	0,015	0,212
Porphyromonadaceae	1,66%	1,28%	0,563	0,716

Prevotellaceae	6,21%	12,10%	0,158	0,592
Rikenellaceae	1,41%	2,01%	0,458	0,618
Clostridiales;Other	0,65%	1,29%	0,313	0,613
Clostridiales;f__	7,51%	3,62%	0,038	0,274
Clostridiaceae	2,10%	2,01%	0,953	0,991
Lachnospiraceae	26,63%	18,36%	0,032	0,265
Ruminococcaceae	30,24%	16,92%	<0,001	0,024
Veillonellaceae	1,90%	3,80%	0,096	0,449
Erysipelotrichaceae	1,14%	0,47%	0,248	0,649
Alcaligenaceae	0,32%	1,13%	<0,001	0,023
Enterobacteriaceae	0,22%	1,05%	0,136	0,539
Verrucomicrobiaceae	0,99%	2,01%	0,602	0,751

Supplementary Table 3. Differences in microbial abundance at the phylum level in saliva samples. Statistical analysis: Welch's t-test with Benjamini-Hochberg FDR multiple test correction.

	**Control Subjects**	**Lynch Patients**	**p value**	**q value**
Unassigned bacteria	3,53%	1,76%	<0,001	0,002
Actinobacteria	2,13%	4,44%	0,001	<0,001
Bacteroidetes	22,27%	25,53%	0,285	0,488
Firmicutes	18,69%	33,17%	0,000	<0,001
Fusobacteria	3,04%	4,74%	0,023	0,11
Proteobacteria	48,18%	28,44%	0,001	<0,001

Supplementary Table 4. Differences in microbial abundance at the family level in saliva samples. Statistical analysis: Welch's t-test with Benjamini-Hochberg FDR multiple test correction.

	**Control Subjects**	**Lynch Patients**	**p value**	**q value**
Unassigned bacteria	3,53%	1,76%	<0,001	0,011
Actinomycetaceae	0,57%	1,22%	0,004	0,11
Micrococcaceae	1,31%	2,76%	0,017	0,187
Porphyromonadaceae	6,89%	4,32%	0,111	0,582
Prevotellaceae	9,66%	16,73%	0,002	0,069
[Paraprevotellaceae]	3,94%	3,18%	0,340	0,568
Gemellaceae	1,67%	1,77%	0,936	1,028
Carnobacteriaceae	0,82%	1,54%	0,005	0,116
Streptococcaceae	6,71%	9,96%	0,027	0,22
Lachnospiraceae	1,22%	0,49%	0,019	0,196
Ruminococcaceae	1,32%	0,06%	0,011	0,133
Veillonellaceae	5,93%	17,95%	<0,001	<0,001
Fusobacteriaceae	2,53%	3,42%	0,131	0,626
Leptotrichiaceae	0,50%	1,32%	0,001	0,026
Burkholderiaceae	0,91%	1,04%	0,690	0,867
Neisseriaceae	22,38%	14,71%	0,105	0,57
Campylobacteraceae	0,96%	1,02%	0,746	0,901
Pasteurellaceae	23,61%	11,39%	<0,001	<0,001

Supplementary Table 5. Differences in microbial abundance at the genus level in fecal samples. Statistical analysis: Welch's t-test with Benjamini-Hochberg FDR multiple test correction.

	**Control Subjects**	**Lynch Patients**	**p value**	**q value**
Unassigned bacteria	1,39%	2,71%	0,003	0,135
Bifidobacterium	0,76%	2,16%	0,064	0,508

Bacteroides	13,27%	24,34%	0,015	0,311
Parabacteroides	1,65%	1,28%	0,561	0,716
Prevotella	6,21%	12,10%	0,158	0,695
Rikenellaceae;g__	1,38%	1,98%	0,467	0,627
Clostridiales;Other	0,65%	1,29%	0,313	0,776
Clostridiales;f__;g__	7,51%	3,62%	0,038	0,436
Clostridiaceae;g__	0,96%	1,19%	0,797	0,869
Lachnospiraceae;Other	1,78%	1,97%	0,768	0,847
Lachnospiraceae;g__	7,57%	5,22%	0,062	0,51
Blautia	3,96%	1,72%	0,050	0,501
Coprococcus	3,46%	1,69%	0,001	0,097
Dorea	1,07%	0,55%	0,042	0,467
Lachnospira	4,17%	4,70%	0,707	0,806
Roseburia	2,18%	1,41%	0,349	0,527
[Ruminococcus]	2,28%	0,87%	0,434	0,615
Ruminococcaceae;g__	18,92%	10,33%	0,002	0,14
Faecalibacterium	6,82%	3,25%	0,002	0,114
Ruminococcus	3,39%	2,48%	0,343	0,521
Phascolarctobacterium	0,87%	1,59%	0,198	0,679
Sutterella	0,30%	1,02%	0,001	0,088
Akkermansia	0,99%	2,01%	0,603	0,742

Supplementary Table 6. Differences in microbial abundance at the species level in fecal samples. Statistical analysis: Welch's t-test with Benjamini-Hochberg FDR multiple test correction.

	**Control Subjects**	**Lynch Patients**	**p value**	**q value**
Unassigned bacteria	1,39%	2,71%	0,003	0,114
Bifidobacterium;s__adolescentis	0,57%	1,43%	0,196	0,689
Bacteroides;s__	8,03%	18,73%	0,004	0,1
Bacteroides;s__plebeius	0,78%	1,63%	0,395	0,626
Bacteroides;s__uniformis	2,07%	2,38%	0,732	0,838
Parabacteroides;s__	1,17%	0,88%	0,648	0,794
Prevotella;s__	0,30%	3,95%	0,030	0,302
Prevotella;s__copri	5,36%	8,11%	0,486	0,658
Rikenellaceae;g__;s__	1,38%	1,98%	0,467	0,612
Clostridiales;Other	0,65%	1,29%	0,313	0,758
Clostridiales;f__;g__;s__	7,51%	3,62%	0,038	0,444
Clostridiaceae;g__;s__	0,96%	1,19%	0,797	0,863
Lachnospiraceae;Other	1,78%	1,97%	0,768	0,845
Lachnospiraceae;g__;s__	7,57%	5,22%	0,062	0,566
Blautia;s__	3,94%	1,71%	0,051	0,524
Coprococcus;s__	2,50%	1,51%	0,015	0,339
Dorea;s__	1,02%	0,53%	0,042	0,443
Lachnospira;s__	4,17%	4,70%	0,707	0,82
Roseburia;Other	1,10%	1,01%	0,841	0,91
Roseburia;s__	1,05%	0,39%	0,271	0,576
[Ruminococcus];s__gnavus	1,53%	0,28%	0,366	0,605
Ruminococcaceae;g__;s__	18,92%	10,33%	0,002	0,119
Faecalibacterium;s__prausnitzii	6,81%	3,24%	0,002	0,114
Ruminococcus;s__	3,34%	2,42%	0,334	0,575
Phascolarctobacterium;s__	0,87%	1,59%	0,198	0,695

Sutterella;s__	0,30%	1,02%	0,001	0,037
Akkermansia;s__muciniphila	0,99%	2,01%	0,603	0,725

Supplementary Table 7. Differences in microbial abundance at the genus level in saliva samples. Statistical analysis: Welch's t-test with Benjamini-Hochberg FDR multiple test correction.

	**Control Subjects**	**Lynch Patients**	**p value**	**q value**
Unassigned bacteria	3,53%	1,76%	<0,001	0,036
Actinomyces	0,55%	1,18%	0,005	0,182
Rothia	1,31%	2,76%	0,017	0,235
Porphyromonas	6,50%	4,00%	0,120	0,549
Prevotella	9,66%	16,73%	0,002	0,106
[Prevotella]	3,94%	3,18%	0,341	0,543
Gemellaceae;g__	1,64%	1,69%	0,962	1,059
Granulicatella	0,81%	1,53%	0,005	0,545
Streptococcus	6,68%	9,92%	0,027	0,273
Veillonella	4,84%	16,00%	<0,001	<0,001
Fusobacterium	2,53%	3,42%	0,132	0,563
Leptotrichia	0,50%	1,18%	0,002	0,124
Lautropia	0,90%	1,04%	0,692	0,835
Neisseriaceae;g__	1,22%	1,07%	0,771	0,89
Neisseria	19,87%	13,27%	0,128	0,561
Campylobacter	0,96%	1,02%	0,746	0,882
Pasteurellaceae;Other	1,16%	0,13%	0,151	0,569
Actinobacillus	1,24%	1,44%	0,765	0,889
Aggregatibacter	1,94%	0,95%	0,084	0,843
Haemophilus	19,27%	8,87%	<0,001	0,026

Supplementary Table 8. Differences in microbial abundance at the species level in saliva samples. Statistical analysis: Welch's t-test with Benjamini-Hochberg FDR multiple test correction.

	**Control Subjects**	**Lynch Patients**	**p value**	**q value**
Unassigned bacteria	3,53%	1,76%	<0,001	0,048
Actinomyces;s__	0,55%	1,18%	0,005	0,241
Rothia;s__mucilaginosa	0,81%	1,81%	0,043	0,387
Porphyromonas;s__	6,01%	3,56%	0,137	0,591
Prevotella;s__	1,75%	3,12%	0,002	0,123
Prevotella;s__melaninogenica	5,94%	11,03%	0,008	0,229
Prevotella;s__pallens	0,49%	1,24%	0,015	0,261
[Prevotella];s__	3,39%	2,61%	0,239	0,592
Gemellaceae;g__;s__	1,64%	1,69%	0,962	1,088
Granulicatella;s__	0,81%	1,53%	0,005	0,234
Streptococcus;s__	6,67%	9,85%	0,028	0,319
Veillonella;s__dispar	4,74%	15,85%	<0,001	<0,001
Fusobacterium;s__	2,53%	3,42%	0,132	0,595
Leptotrichia;s__	0,50%	1,18%	0,002	0,141
Lautropia;s__	0,90%	1,04%	0,692	0,863
Neisseriaceae;g__;s__	1,22%	1,07%	0,771	0,924
Neisseria;s__	7,68%	4,90%	0,386	0,569
Neisseria;s__cinerea	11,08%	7,72%	0,129	0,588
Campylobacter;s__	0,96%	1,02%	0,747	0,911
Pasteurellaceae;Other	1,16%	0,13%	0,151	0,611

Actinobacillus;s__parahaemolyticus	1,15%	1,41%	0,694	0,864
Aggregatibacter;s__segnis	1,35%	0,76%	0,151	0,619
Haemophilus;Other	1,57%	0,83%	0,133	0,592
Haemophilus;s__	1,18%	0,53%	0,268	0,616
Haemophilus;s__parainfluenzae	16,49%	7,51%	0,001	0,091
